# Direct Metatranscriptomic Survey of the Sunflower Microbiome and Virome

**DOI:** 10.3390/v13091867

**Published:** 2021-09-18

**Authors:** Ziyi Wang, Achal Neupane, Jiuhuan Feng, Connor Pedersen, Shin-Yi Lee Marzano

**Affiliations:** 1 Department of Biology and Microbiology, South Dakota State University, Brookings, SD 57007, USA; Ziyi.wang@jacks.sdstate.edu (Z.W.); achal.neupane@jacks.sdstate.edu (A.N.); connor.pedersen@jacks.sdstate.edu (C.P.); 2 Department of Agronomy, Horticulture and Plant Science, South Dakota State University, Brookings, SD 57007, USA; srfengjh@lpht.com; 3 United States Department of Agriculture-Agricultural Research Service, Toledo, OH 43606, USA

**Keywords:** sunflower, virome, mycoviruses, microbiome

## Abstract

Sunflowers (*Helianthus annuus* L.) are susceptible to multiple diseases in field production. In this study, we collected diseased sunflower leaves in fields located in South Dakota, USA, for virome investigation. The leaves showed visible symptoms on the foliage, indicating phomopsis and rust infections. To identify the viruses potentially associated with the disease diagnosed, symptomatic leaves were obtained from diseased plants. Total RNA was extracted corresponding to each disease diagnosed to generate libraries for paired-end high throughput sequencing. Short sequencing reads were assembled de novo and the contigs with similarities to viruses were identified by aligning against a custom protein database. We report the discovery of two novel mitoviruses, four novel partitiviruses, one novel victorivirus, and nine novel totiviruses based on similarities to RNA-dependent RNA polymerases and capsid proteins. Contigs similar to bean yellow mosaic virus and Sclerotinia sclerotiorum hypovirulence-associated DNA virus were also detected. To the best of our knowledge, this is the first report of direct metatranscriptomics discovery of viruses associated with fungal infections of sunflowers bypassing culturing. These newly discovered viruses represent a natural genetic resource from which we can further develop potential biopesticide to control sunflower diseases.

## 1. Introduction

Annual sunflower (*Helianthus annuus* L.) production is an economically important crop. In China, the total sunflower acreage planted, mainly for the non-oilseed types, was relatively stable with fluctuations from 1978 to 2018. The average yield, however, increased steadily from 1077 kg/ha to 2707 kg/ha, so the overall trend of sunflower production is increasing. In the USA, the yield and production show a stable trend due to the improved hybrid varieties, despite declining sunflower acreage [1]. Both oil and confection sunflower are prone to several major diseases, such as downy mildew, phomopsis stem canker, and rust, which can greatly impact the yield. The causal agents of these diseases are *Plasmopara halstedii*, *Phomopsis helianthi*, and *Puccinia helianthi*, respectively, and symptoms are readily identifiable in the field. Unfortunately, existing controls based on host resistance or fungicides have not provided sufficient control to date, which calls for developing alternative control methods. 

Recent surveys have demonstrated that plants often harbor novel mycoviruses [2], which have the potential to be developed for biopesticides [3] if hypovirulence (reduced virulence) in the fungal hosts is found. Hypovirulence in fungi has been reported in many different fungi, such as *Cryphonectria parasitica*, *Sclerotinia sclerotiorum*, *Rhizoctonia* spp., and *Fusarium* spp., etc., although many mycoviruses appear to cause asymptomatic infections (reviewed in [4,5,6]). Field studies based on foliar sprays or “bio-priming” seed treatment of mycelial homogenates of fungal isolates infected with mycoviruses showed significantly reduced severity of fungal diseases on brassica oilseed crops [7,8]. However, few potential mycoviruses associated with sunflower fungal pathogens have been discovered and characterized with only a few focused on the oomycete *P. halstedii* [9,10], and none for *Phomopsis helianthi* and *Puccinia helianthi*. Besides their virocontrol aspect, past surveys have observed that mycoviruses of the same family infecting the same fungal host tend to cluster closely phylogenetically [11] and can be useful for understanding fungal epidemiology [12]. Cryptic and persistent plant-associated viruses also have been found to change plant volatiles and protect the plant host from aphids, a common vector for acute plant viruses [13]. Therefore, the presence of specific viruses may provide useful information to predict the incidence of fungal or plant viral diseases. Currently, there are still limitations on the resolution of the fungal nuclear ribosomal internal transcribed spacer (ITS) region to make an accurate identification of some fungal endophytes, so plant-associated viromes could serve as biomarkers for the identification of the fungal endophyte community. 

Sometimes, lab isolates of fungal cultures have sectoring growth and are discarded by the pathologists when the transferred isolates grow slowly and become hard to maintain. Therefore, candidates of biopesticide or biotechnological use could be eliminated in the culturing/serial passage process, e.g., *Macrophomina phaseolina tobamo*-like virus 1 [11,14]. Moreover, some fungal pathogens, e.g., sunflower downy mildew and rust, being obligate parasites, cannot be cultured in the lab. Metatranscriptome surveys of plant leaves/roots or fungal isolates of plant pathogens have allowed for the discovery of many novel mycoviruses [2,15] bypassing the culturing step. Once potential mycoviruses are prioritized for further characterization, the resulting mycoviruses can sometimes reprogram the pathogenic fungi to grow as beneficial endophytes to increase yield beyond the protective effect [16].

In this study, we directly identify novel viruses from the diseased tissues of sunflowers caused by rust and phomopsis. This direct approach of sequencing diseased tissue without first culturing bypasses the possibility of selecting for the mildly attenuating or asymptomatic mycovirus infections and allows us to characterize the plant viromes of the pathogens to include the ones that cannot be easily cultured in the lab.

## 2. Materials and Methods

### 2.1. Field Sampling and Sample Processing

To identify potential mycoviruses from the field, tissue samples of diseased sunflowers were collected in the summer of 2017. Symptomatic leaf samples from four diseased plants, each for phomopsis and rust, from Brookings County and Sully County in South Dakota, USA, were sequenced, respectively, in this study. The two diseased plant samples were collected separately from different fields on different dates and were not coinfected by the two pathogens. The phomopsis plants were sampled early to mid-September 2017 for leaves showing symptoms but not yet completely wilted. Specifically, the green leaf tissues on the edge of the lesions caused by phomopsis were sampled because the lesion area had very little intact RNA left (data not shown). Four leaves per plant were stacked, punched into discs, divided into 150 mg per tube, flash-frozen in liquid nitrogen, and stored at −80 ℃ until further processing. Leaf discs containing rust pustules and between the pustules were both included in the RNA extraction.

### 2.2. RNA Extraction

RNA extraction was conducted as previously described by Marzano et al. [11]. Leaf discs were ground in tubes dipped in liquid nitrogen by metal beads, and total RNA was extracted using Qiagen RNeasy Plant Mini Kit following the manufacturer’s instruction (Valencia, CA, USA). RNA samples were treated with DNase I and evaluated for integrity by agarose gel electrophoresis.

### 2.3. Library Construction

Total RNA (approximately 1 μg) free of genomic DNA was depleted of rRNAs with the Ribo-Zero Plant Kit and used as a template to construct paired-end libraries with a ScriptSeq RNA sample preparation kit (both manufactured by Illumina, San Diego, CA, USA), and were cleaned and sequenced on an Illumina HiSeq 4000 for pair-end sequencing through the W.M. Keck Center, University of Illinois. SF1 and SF2 libraries were constructed using RNA extracts from phomopsis- and rust-infected leaves, respectively.

### 2.4. Sequence Analysis

The microbiome associated with sunflower leaves was profiled using DIAMOND [17] by aligning the short reads to the National Center for Biotechnology Information (NCBI) nonredundant (nr) amino acid sequence database, parsed, and assigned to taxa using MEGAN 6 [18]. The RNA-Seq library reads were analyzed and uploaded to the Sequence Read Archive (SRA) of the National Center for Biotechnology Information (NCBI) under accession PRJNA657097. There were 65,156,534 and 114,034,206 reads from SF1 (SRS7200872) and SF2 (SRR12450951) libraries, respectively. Adapters were first trimmed using bbduk (JGI) and assembled into contigs using a TRINITY (v2.5.1) de novo transcriptome assembler [19]. After trimming of the adapters, there were ~98% of reads left for assembly. Contigs with significant similarity of viral amino acid sequences were identified using USEARCH ublast [20] with a parameter e-value of 0.0001 for significant hits and compared to a custom database containing sunflower and viral amino acid sequences from GenBank using BLASTX to exclude misidentified sequences.

The predicted amino acid sequences of potential novel viruses were aligned to the contigs from NCBI using ClustalX 2.0 [21]. Phylogenetic trees were generated with RAxML [22] and modified with Dendroscope 3.7.2 [23]. Poorly supported lineages were contracted. To better present the evolutionary relationships between the novel viruses and other known viruses, the minimum support thresholds were set to 47 for *Partitiviridae* and 44 for *Mitoviridae* because the new viral contigs would not cluster with other viruses otherwise. For other viruses, the thresholds were set at 50.

## 3. Results

### 3.1. Metatranscriptomic Comparison of Microbiome/Virome from Two Libraries

**Figure 1:**
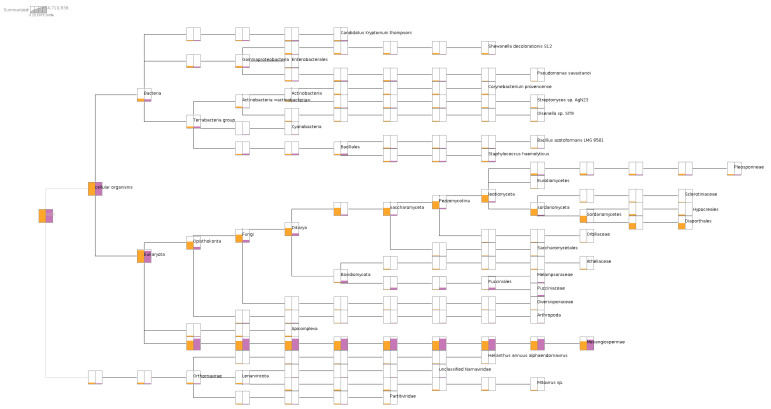
Metatranscriptomic comparison of two libraries showing the microbiome/virome composition in libraries SF1 and SF2. Both libraries contained more than 60% of sunflower transcripts under Mesangiospermae clade. Only library SF1, labeled in orange, had reads from Diaporthales for phomopsis disease, whereas only library SF2 contained reads from Pucciniaceae for rust disease.

Upon aligning the short reads to NCBI nr-database, the metatransciptomic comparison of the two libraries using MEGAN analysis provided verification of the association with their respective plant diseases. The causal agent(s) of phomopsis stem canker of sunflower were detected from infected leaves, coinfected by other fungi under families Sclerotiniaceae, Hypocreales, and Pleosporineae. Some bacterial taxa are common to both libraries in approximately the same amount, including *Candidatus kryptonium*, *Bacillus azotoformans*, and *Staphylococcus haemolyticus*. However, the SF1 library had nearly 10X more Actinobacteria in normalized read counts than the SF2 library. In addition, most of the HaEV1 reads were aligned to the SF1 library, in contrast to the SF2 library, which had only mitoviruses and dsRNA viruses (Durnavirales) detected using the MEGAN analysis based on short read alignments (Figure 1).

**Figure 2:**
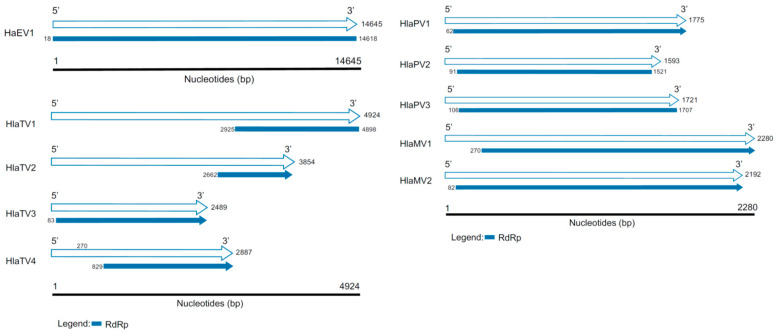
Genome organizations of the viruses discovered in this study. The white arrows represent the length of the contigs discovered in this study, and the RNA-dependent RNA polymerases (RdRps) are labeled in blue. The blue blunt ends represent complete RdRps, and the blue arrows indicate the RdRps are partial.

Table A1 summarizes all the viruses discovered in this study with identities to RNA-dependent RNA polymerases (RdRps) or capsid proteins (CPs). Based on the RdRp identities and contig lengths, the virus genomes included in this report are one endornavirus, two mitoviruses, three partitiviruses, and four totiviruses (Figure 2). Based on the similarities to CPs, 15 viral contigs were appended in Table A1 as well. Based on both RdRps and CPs, three known viruses, including Helianthus annuus alphaendornavirus 1 (HaEV1), bean yellow mosaic virus, and a short contig of Sclerotinia sclerotiorum hypovirulence-associated DNA virus were detected. Four of the viral contigs likely had complete RdRps, including Helianthus annuus alphaendornavirus (HaEV1), Helianthus annuus leaf-associated partitivirus 2 (HlaPV2), Helianthus annuus leaf-associated partitivirus 3 (HlaPV3), and Helianthus annuus leaf-associated totivirus 1 (HlaTV1).

### 3.2. Endornavirus Genome

The predicted amino acid sequence of a known virus, HaEV1, (14,645 nt; MT873524) contained a conserved polyprotein with 99.8% identity to the previously published Helianthus annuus alphaendornavirus sequence (NC_040799.1) and is related to another alphaendornavirus (MN015676.1) with 40% identity. Besides these two viruses, the HaEV1 amino acid sequence was also grouped with other endornaviruses, including Helicobasidium mompa alphaendornavirus 1, Soybean leaf-associated endornavirus 1, Phytophthora endornavirus 1, and Vicia faba endornavirus. Therefore, based on the structure of the phylogenetic tree and the genus of known viruses from the International Committee on Taxonomy of Viruses (ICTV), HaEV1 belongs to *Alphaendornavirus* genus.

### 3.3. Putative Mitovirus Genome

**Figure 3:**
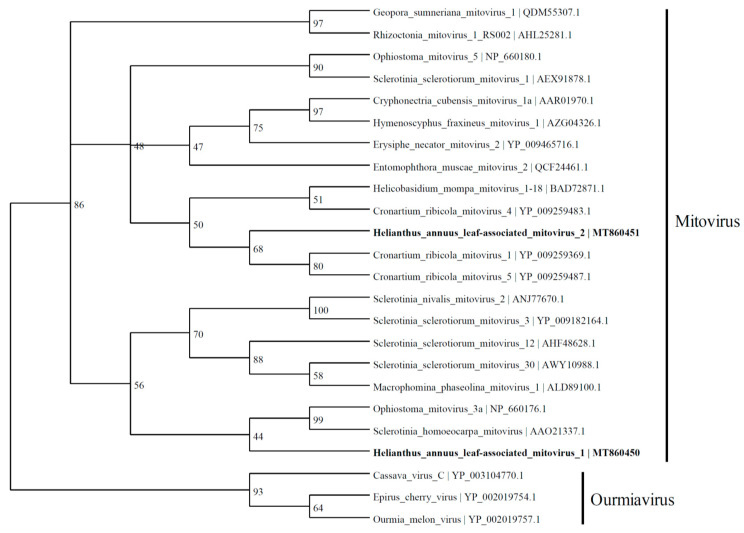
Phylogenetic tree depicting the relationships of the predicted amino acid sequence of RNA-dependent RNA polymerase of *Helianthus annuus* leaf-associated mitovirus 1 and *Helianthus annuus* leaf-associated mitovirus 2 with other confirmed and proposed members of the family *Mitoviridae* and genus *Ourmiavirus*.

HlaMV1 (2280 nt; MT860450), discovered in this study, contained an RdRp with 43.7% identity to the RdRp-like protein of *Sclerotinia homoeocarpa mitovirus* (2632 nt; AY172454.1) and 43.9% identity to the RdRp of *Ophiostoma mitovirus* 3a (2617 nt) [24] in the *Mitovirus* genus (Figure 3). HlaMV2 (2192 nt; MT860451) has similarities to the *Cronartium ribicola mitovirus* 5 (2631 nt; NC_030399.1) and *Cronartium ribicola* mitovirus 1 (2715 nt; NC_030393.1).

### 3.4. Putative Partitivirus Genome

**Figure 4:**
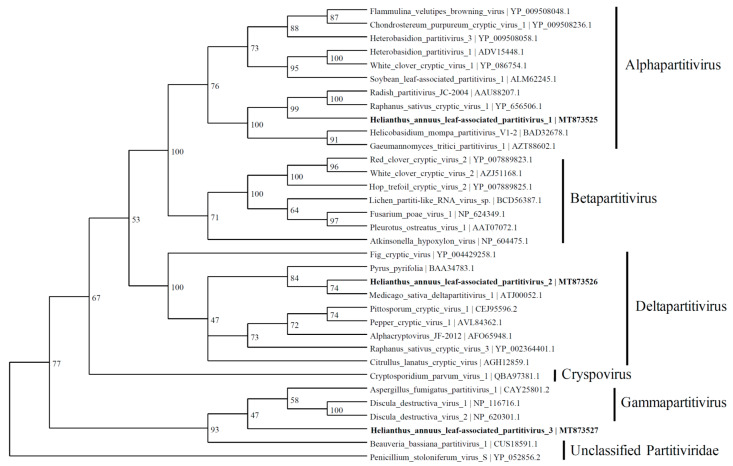
Phylogenetic tree depicting the relationships of the predicted amino acid sequence of RNA-dependent RNA polymerase of the *Helianthus annuus* leaf-associated partitivirus 1, the *Helianthus annuus* leaf-associated partitivirus 2, and the *Helianthus annuus* leaf-associated partitivirus 3 with other confirmed and proposed members of the family *Partitiviridae*.

Three contigs were predicted to express the RdRp proteins with similarities to the viruses belonging to the *Partitiviridae* family. The contig named HlaPV1 (1775 nt; MT873525) contained an incomplete open reading frame (ORF) of RdRp, which shares identity with Raphanus sativus cryptic virus 1 (66.8%) (YP_656506.1), *Radish partitivirus* JC-2004 (66.1%) (AAU88207.1), and many other alphapartitiviruses (Figure 4). The RdRp expressed by a contig named *Helianthus annuus* leaf-associated partitivirus 2 (HlaPV2; 1593 nt; MT873526) shows similarities to the RdRp of Medicago sativa deltapartitivirus 1 (MF443259.1) and *Pyrus pyrifolia* associated RNA virus (BAA34783.1) with 60.5% and 59.7% identity, respectively. The Helianthus annuus leaf-associated partitivirus 3 (HlaPV3; 1721 nt; MT873527) contig shares 76% identity with unclassified Partitiviridae: Verticillium dahlia partitivirus 1 and *Botryotinia fuckeliana partitivirus* 1 (NC_010349.1) through BLASTX. The phylogenetic tree shows that HlaPV3 was most closely related to members of the *Gammapartitivirus* genus (Figure 4).

**Figure 5:**
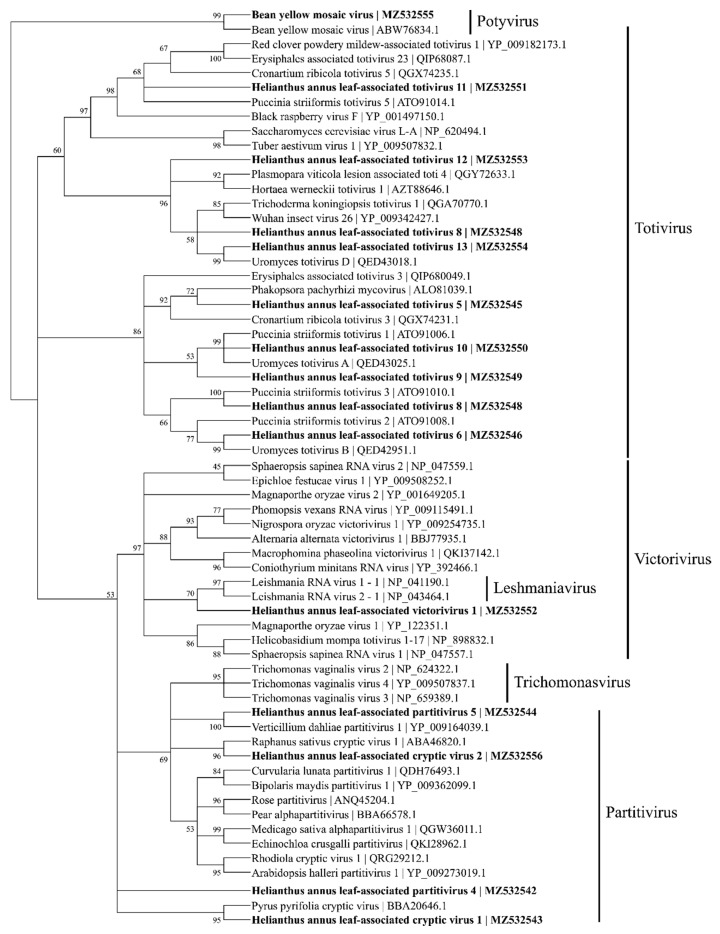
Phylogenetic tree based on maximum likelihood depicting the relationships of the predicted amino acid sequences of coat proteins for 14 viral contigs belonging to Totiviridae and Parititiviridae using a detected bean yellow mosaic virus as the outgroup. A total of 1000 bootstrap replicates were analyzed.

Based on the coat protein identities, four viral contigs were identified to be within Partitiviridae and named as *Helianthus annus* leaf-associated partitivirus 4 (MZ532542), *Helianthus annus* leaf-associated cryptic virus 1 (MZ532543), *Helianthus annus* leaf-associated partitivirus 5 (MZ532544), and Helianthus annus leaf-associated cryptic virus 2 (MZ532556) (Figure 5).

### 3.5. Putative Totivirus Genome

Based on the similarity to RdRp, four viral contigs with similarities to the *Totiviridae* family were found and named as *Helianthus annuus* leaf-associated totivirus 1 (4924 nt; MT873528), *Helianthus annuus* leaf-associated totivirus 2 (3854 nt; MT873529), *Helianthus annuus* leaf-associated totivirus 3 (2489 nt; MT873530), and *Helianthus annuus* leaf-associated totivirus 4 (2887 nt; MT873531). The predicted amino acid sequence of HlaTV1 contains a complete RdRp which shares 50% identity to *Puccinia striiformis totivirus* 4 (KY207364). The HlaTV2 contig has 37% identity with RdRp of *Puccinia striiformis totivirus* 2 (KY207362). Both HlaTV3 and HlaTV4 are similar to *Puccinia striiformis totivirus* 5 (KY207365) with identities of 49% and 79%, respectively. It is worth noting that *Puccinia striiformis* is another rust fungus, which suggests that our approach was successful in identifying viruses associated with the pathogenic fungi. These four viral contigs are more closely related to the viruses belonging to the *Totivirus* genus than other genera in the *Totiviridae* family (Figure 5). According to the reconstructed phylogenetic tree of *Totiviridae*, HlaTV1 with HlaTV2 and HlaTV3 with HlaTV4 clustered separately, which indicates that HlaTV1 and HlaTV2 are more closely related and HlaTV3 and HlaTV4 are more related phylogenetically.

**Figure 6:**
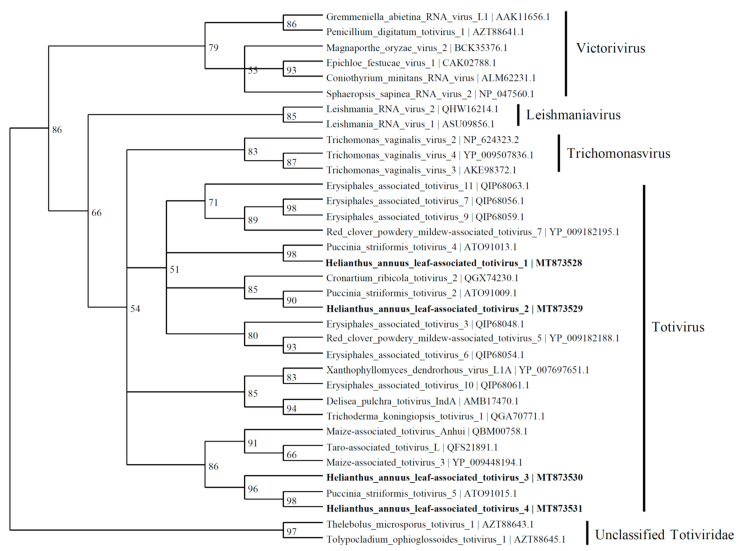
Phylogenetic tree depicting the relationships of the predicted amino acid sequence of the RNA-dependent RNA polymerase of *Helianthus annuus* leaf-associated totivirus 1, *Helianthus annuus* leaf-associated totivirus 2, *Helianthus annuus* leaf-associated totivirus 3, and *Helianthus annuus* leaf-associated totivirus 4 with other confirmed and proposed members of the family *Totiviridae*.

Based on the similarity to coat protein (CP), 10 viral contigs were identified, including 9 totiviruses and a victorivirus (MZ532545~54) (Figure 6). Among the partitivirus contigs, HlaPV3 and HlaPV5 could be the two RNA segments for the same virus based on the fact that the majority of the reads both came from the SF1 library. In addition, contigs of HlaPV2 and HlaCV1 could come from the same virus too, based on the same read count ratios between the reads from SF1 and SF2 libraries.

A potyvirus-like contig was also identified, which shares 99.5% amino acid identity to bean yellow mosaic virus (BYMV) for the 555 nt contig and has only 70% nucleotide identity to the Meadow saffron breaking virus polyprotein gene and the Butterfly flower mosaic virus. As the sequences for BYMV are very diverse, and one of the top hits (93.3% nt identity) in BLASTX for this BYMV-like sequence is a BYMV isolate from sunflowers in Iran encoding a portion of the CP, we still named it as BYMV. The naming is based on the knowledge that CPs need to form specific structures to assemble virus particles, so their amino acid sequences are often highly conserved. Moreover, based on coat protein similarity, we detected a short contig (197 nt) that came from very few reads in both libraries and is similar to the Sclerotinia sclerotiorum hypovirulence-associated DNA virus. It is divergent from the other detected CPs from dsRNA viruses, and therefore excluded from the phylogenetic analysis shown in Figure 6. More characterization of the whole genome sequence is needed in future studies.

## 4. Discussion

In this study, we identified 25 putative viral contigs using a metatranscriptomics approach, ranging from 1.6 kb to 14.6 kb for the RNA viruses, and one short DNA virus contig. Only one of the viruses, an endornavirus, had been described previously as Helianthus annuus alphaendornavirus (HaEV1). It was sampled from leaves showing typical symptoms of brown, irregular-shaped spots on infected leaves, which coalesced into large patches of dead tissue spreading in from the leaf margin, indicating *Phomopsis* spp. infection. *Phomopsis* stem canker in sunflowers has been reported to be associated with at least 15 species of *Diaporthe* that are either pathogenic or non-pathogenic [25] and can be a complex of the pathogenic *Diaporthe* (*Phomopsis*) species [26]. Although this virus is thought to exist without having direct associations to fungi, our detection of it being associated with *Phomopsis* warrants further investigation into whether the virus replicates in *P. helianthin,* and the effects of HaEV1 on fungal virulence. It has been reported that a plant virus, cucumber mosaic virus, can naturally infect a fungal host, *Rhizoctonia solani* [27]. Therefore, it will be interesting to verify whether it is the same for HEV1, especially since we have detected contigs of HEV1 in a separate metatranscriptome (deposited under NCBI PRJNA753120) directly from *Diaporthe spp*., which will be reported in a separate paper. The current study has the limitation that more validation is required to cross-check with *Diaporthe metatranscriptomes*, for example, to make a connection to phomopsis-infecting mycoviruses. Nevertheless, this study profiled known and novel sunflower-associated viruses from the field and provided hypotheses for further laboratory determination. In addition, endornaviruses belong to a group of viruses without true virions, which will require the construction of infectious cDNA clones driven by a T7 promoter in future studies to establish cause-and-effect relationships.

Based on RdRps, totiviruses with less than 50% amino acid sequence identity are considered different species. Therefore, HlaTV2 and HlaTV3 are considered new species. For partitiviruses, the threshold is 40%: amino acid sequence similarity >40% between RdRps of viruses from different species in the same phylogenetic cluster and <40% between members of species in different clusters; therefore, new species are reported in this study. Based on CPs, we discovered more viral contigs than RdRps, and it will require further investigation to better characterize these newly reported viruses. Using similarities to existing CPs, we detected more contigs, probably because they need to be translated to specific protein structures to assemble virions and are more conserved. Although some viruses lack CPs, such as endornaviruses and mitoviruses, using CPs to detect new viruses is very useful for the viruses that encode CPs.

We previously reported a survey of soybean leaf-associated virome from field-collected tissues regardless of the disease symptoms [2]. In this study, we instead focused only on diseased tissues because of the potential to link the diseases with the viruses. Even though specific non-pathogenic viruses are associated with tissues infected by pathogenic fungi, it does not necessarily mean that these viruses do not cause hypovirulence of the fungal pathogens. Potential hypovirulence-associated mycoviruses discovered in phytobiomes [2,3] can be rescued and reintroduced back to the field in a high viral titer after culturing in the lab. To build up viral titer, once the virus is rescued by reverse genetics approaches [15] or purified along with the fungal isolates [7], abnormal growth of the fungal hyphae should be noted and preferentially selected for serial transfer. Rather than following the traditional method of maintaining a fungal isolate in mycology labs, the abnormal-looking portion of older growth, specifically the fuzzy, sectoring, and oddly pigmented part of the plate, should be selected for transfer. Once the viral titer is increased after repeated lab conditioning, an inoculum can be obtained from a culture with even growth. For example, a stable culture of *S. sclerotiorum* that does not produce sclerotia is a desirable phenotype [3], and the hypovirulence strain can be introduced directly to the field. Furthermore, crude extraction of the virions can be achieved by low-speed centrifugation and used for virocontrol development for either agricultural purposes [28] or to be explored for medical uses [29]. Again, the exact hosts of these viruses reported in the current study require further validation, and the effects on their hosts are yet to be determined.

To simultaneously compare the changes in microbiome and virome in the field or in greenhouse production, the same direct sequencing of total RNA without culturing, in a repeated sampling scheme, should be carried out in the future. Ecological modeling should be performed to include the abundances of each virus and overall microbial population, including fungal pathogens, shown in normalized read counts based on the high throughput sequencing data, in conjunction with the disease severity rating, and the environmental conditions for multiple time points. Applying such a systems approach will provide us new insights into the emergence and maintenance of viromes facing environmental variability, which would be proactive, considering their importance as ecological drivers of the pathogen population and overall microbial composition. A study to include multiple sites can be advantageous, especially if the disease in question is widespread and the pathogen is cosmopolitan. It would be interesting to monitor whether there is a correlation between the mycoviral read counts and the disease severities and yield intra-annually [30], since yield enhancement has been reported in a mycovirus that presents in fields of brassica spp. [16]. As global climate change and extreme weather intensify, exploring virome-mediated pathogen control and plant growth promotion in agricultural production is urgently needed more than ever. The metatranscriptomics approach used in this study provides an unbiased snapshot of the plant-associated virome, including some potentially beneficial mycoviruses.

## Data Availability

All data generated and analyzed during this study are included in this article.

## Appendix A

**Table A1:** Novel mycoviruses discovered by metatranscriptomics approach from sunflower (RdRp: RNA-dependent RNA polymerase). The SF1 library was constructed from RNA extracts of phomopsis-infected leaves, while the SF2 library was constructed from rust-infected leaves.

Contig Name	SF 1 Read Counts	SF 2 Read Counts	Abbreviation	Genbank Accession	Genome	Contig Length (nt)	Identity (%)	Putative Function (Most Similar Virus)
Helianthus annuus alphaendornavirus	16,590	36	HaEV1	MT873524	ss(+) RNA	14645	99.8	RdRp, Helianthus annuus alphaendornavirus
Helianthus annuus leaf-associated mitovirus 1	208,030	130	HlaMV1	MT860450	ss(+) RNA	2280	43.9	RdRp, Ophiostoma_mitovirus_3a
Helianthus annuus leaf-associated mitovirus 2	136	8446	HlaMV2	MT860451	ss(+) RNA	2192	36.2	RdRp, Cryphonectria_parasitica_mitovirus_1-NB631
Helianthus annuus leaf-associated partitivirus 1	5528	6816	HlaPV1	MT873525	dsRNA	1775	66.8	RdRp, Raphanus sativus cryptic virus 1
Helianthus annuus leaf-associated partitivirus 2	11,560	13,174	HlaPV2	MT873526	dsRNA	1593	60.5	RdRp, Medicago sativa deltapartitivirus 1
Helianthus annuus leaf-associated partitivirus 3	10,340	8	HlaPV3	MT873527	dsRNA	1721	76	RdRp, Verticillium dahlia Partitivirus 1
Helianthus annuus leaf-associated totivirus 1	12	3867	HlaTV1	MT873528	dsRNA	4924	50	RdRp, Puccinia striiformis totivirus 4
Helianthus annuus leaf-associated totivirus 2	4	1502	HlaTV2	MT873529	dsRNA	3854	37	RdRp, Puccinia striiformis totivirus 2
Helianthus annuus leaf-associated totivirus 3	0	540	HlaTV3	MT873530	dsRNA	2489	49	RdRp, Puccinia striiformis totivirus 5
Helianthus annuus leaf-associated totivirus 4	2	580	HlaTV4	MT873531	dsRNA	2887	79	RdRp, Puccinia striiformis totivirus 5
H_annus_leaf-associated_partitivirus_4	23	8	HlaPV4	MZ532542	dsRNA	381	67.5	CP; Echinochloa crusgalli partitivirus
H_annus_leaf-associated_cryptic_virus_1	14,353	16,428	HlaCV1	MZ532543	dsRNA	1447	41.1	CP; Pyrus pyrifolia cryptic virus
H_annus_leaf-associated_partitivirus_5	7020	3	HlaPV5	MZ532544	dsRNA	1625	67.6	CP; Verticillium dahliae partitivirus 1
H_annus_leaf-associated_totivirus_5	16	3083	HlaTV5	MZ532545	dsRNA	3121	39.8	CP; Cronartium ribicola totivirus 3
H_annus_leaf-associated_totivirus_6	0	1065	HlaTV6	MZ532546	dsRNA	2907	75.6	CP; Uromyces totivirus B
H_annus_leaf-associated_totivirus_7	23	1468	HlaTV7	MZ532547	dsRNA	1965	39.3	CP; Totiviridae sp.
H_annus_leaf-associated_totivirus_8	18	1826	HlaTV8	MZ532548	dsRNA	2061	62.5	CP; Puccinia striiformis totivirus 3
H_annus_leaf-associated_totivirus_9	6	719	HlaTV9	MZ532549	dsRNA	2536	36.6	CP; Puccinia striiformis totivirus 1
H_annus_leaf-associated_totivirus_10	8	873	HlaTV10	MZ532550	dsRNA	2380	65.2	CP; Puccinia striiformis totivirus 1
H_annus_leaf-associated_totivirus_11	9	417	HlaTV11	MZ532551	dsRNA	2189	45.9	CP; Cronartium ribicola totivirus 5
H_annus_leaf-associated_victorivirus_1	0	203	HlaVV1	MZ532552	dsRNA	1263	41.3	CP; Alternaria alternata victorivirus 1
H_annus_leaf-associated_totivirus_12	4	671	HlaTV12	MZ532553	dsRNA	1709	40.6	CP; Totiviridae sp.
H_annus_leaf-associated_totivirus_13	18	386	HlaTV13	MZ532554	dsRNA	1613	83.6	CP; Uromyces totivirus D
Bean yellow mosaic virus	13	14	HlaPoV1	MZ532555	ssRNA	555	99.5	CP; Bean yellow mosaic virus
H_annus_leaf-associated_cryptic_virus_2	1217	812	HlaCV2	MZ532556	dsRNA	1585	46.1	CP; Raphanus sativus cryptic virus 1
Sclerotinia sclerotiorum hypovirulence-associated DNA virus 1-like virus	2	4	SsHADV-1 like	N/A	ssDNA	197	96	CP, Sclerotinia scleoritorum hypovirulence-associated DNA virus 1
